# The Use of Dexmedetomidine in the Emergency Department: A Cohort Study

**DOI:** 10.5811/westjem.2021.4.50917

**Published:** 2021-08-22

**Authors:** Joseph Sinnott, Christopher V. Holthaus, Enyo Ablordeppey, Brian T. Wessman, Brian W. Roberts, Brian M. Fuller

**Affiliations:** * Washington University School of Medicine in St. Louis, Department of Emergency Medicine, St. Louis, Missouri; † Washington University School of Medicine in St. Louis, Department of Anesthesiology, St. Louis, Missouri; ‡ Cooper University Hospital, Department of Emergency Medicine, Camden, New Jersey

## Abstract

**Introduction:**

Management of sedation, analgesia, and anxiolysis are cornerstone therapies in the emergency department (ED). Dexmedetomidine (DEX), a central alpha-2 agonist, is increasingly being used, and intensive care unit (ICU) data demonstrate improved outcomes in patients with respiratory failure. However, there is a lack of ED-based data. We therefore sought to: 1) characterize ED DEX use; 2) describe the incidence of adverse events; and 3) explore factors associated with adverse events among patients receiving DEX in the ED.

**Methods:**

This was a single-center, retrospective, cohort study of consecutive ED patients administered DEX (January 1, 2017–July 1, 2019) at an academic, tertiary care ED with an annual census of ~90,000 patient visits. All included patients (n= 103) were analyzed for characterization of DEX use in the ED. The primary outcome was a composite of adverse events, bradycardia and hypotension. Secondary clinical outcomes included ventilator-, ICU-, and hospital-free days, and hospital mortality. To examine for variables associated with adverse events, we used a multivariable logistic regression model.

**Results:**

We report on 103 patients. Dexmedetomidine was most commonly given for acute respiratory failure, including sedation for mechanical ventilation (28.9%) and facilitation of non-invasive ventilation (17.4%). Fifty-four (52.4%) patients experienced the composite adverse event, with hypotension occurring in 41 patients (39.8%) and bradycardia occurring in 18 patients (17.5%). Dexmedetomidine was stopped secondary to an adverse event in eight patients (7.8%). Duration of DEX use in the ED was associated with an increase adverse event risk (adjusted odds ratio, 1.004; 95% confidence interval, 1.001, 1.008).

**Conclusion:**

Dexmedetomidine is most commonly administered in the ED for patients with acute respiratory failure. Adverse events are relatively common, yet DEX is discontinued comparatively infrequently due to adverse events. Our results suggest that DEX could be a viable option for analgesia, anxiolysis, and sedation in ED patients.

## INTRODUCTION

The management of sedation, analgesia, and anxiolysis are critically important principles in the emergency department (ED). Dexmedetomidine (DEX) is a centrally acting and selective alpha-2 adrenoreceptor agonist, which inhibits norepinephrine release by binding to presynaptic alpha-2 receptors. It provides sedation, anxiolysis, and analgesia via receptors in the brainstem and spinal cord.^1^,^2^ Furthermore, DEX does not cause respiratory depression, making it an attractive agent for the management of multiple patient populations.

In patients with acute respiratory failure, data from mechanically ventilated intensive care unit (ICU) patients have demonstrated improved outcomes with DEX, when compared to benzodiazepines, including a reduction in delirium and ventilator duration.^2^–^4^ In ICU patients who cannot tolerate non-invasive positive pressure ventilation (NIPPV), DEX has been shown to be effective at facilitating NIPPV and may be associated with improved outcomes (ie, reduced intubation rates and ICU length of stay).^1^,^5^ However, there is a lack of data from the ED domain regarding DEX use in patients with acute respiratory failure. Other descriptions of DEX use in the ED include alcohol withdrawal and procedural sedation. Although the data are limited, a few studies have shown that DEX may reduce the need for endotracheal intubation in patients with alcohol withdrawal, and be a safe and effective procedural sedation agent.^6^–^9^

Given the lack of data and trials regarding DEX use in the ED, there is a significant knowledge gap and lack of familiarity regarding the use of this agent. Furthermore, as DEX has consistently been shown to increase the incidence of hypotension and bradycardia, its safety profile in the ED during routine use is unknown as well. We conducted this study with several objectives in mind: 1) to characterize the use of DEX in the ED; 2) describe the incidence of adverse events in the ED population; and 3) explore factors associated with adverse events among patients receiving DEX in the ED.

## METHODS

### Study Design

This was a single-center, retrospective, cohort study and is reported in accordance with the Strengthening the Reporting of Observational Studies in Epidemiology (STROBE) Statement (see supplemental Table S1). 10 The study was approved by the Human Research Protection Office at the principal investigator’s institution with waiver of informed consent. There was no financial support or funding organization associated with the study.

### Study Setting and Population

The study was conducted at an academic, university-affiliated teaching hospital with an annual ED census of approximately 90,000 patient visits. Given the clinical outcome data regarding DEX, an order-set and protocol was introduced in the ED in 2017. This protocol advocated for a static DEX dose of 0.4 micrograms/kilogram/hour (mcg/kg/hour) in non-intubated patients. In mechanically ventilated patients, the protocol advocated for a starting dose of 0.7 mcg/kg/hour, with a recommended titration of 0.1 mcg/kg/hour every 45 minutes, up to a maximum dose of 1.5 mcg/kg/hour. Titration was by physician order, and not titratable by the nurse. Bolus doses of DEX were not recommended by the protocol, nor given during the study period. Over a 30-month period (January 1, 2017–July 1, 2019), all consecutive patients with an order to receive DEX were identified via electronic health record (EHR) query and were eligible for inclusion. Inclusion criteria were 1) age ≥ 18 years; and 2) the receipt of DEX in the ED for any indication.

**Population Health Research Capsule**

What do we already know about this issue?

*Dexmedetomidine (DEX) provides sedation, anxiolysis, and analgesia and is effective in various clinical situations. However, data is sparse from the emergency department (ED) domain*.

What was the research question?

*How is DEX used in the ED, and what is the incidence of adverse events associated with its use?*

What was the major finding of the study?

*Dexmedetomidine is used primarily in respiratory failure (46.3% of cases). While adverse events are common (52.4% of cases), they are of questionable clinical significance*.

How does this improve population health?

*The use of dexmedetomidine could be an important adjunct in the care of multiple patient cohorts in the ED*.

### Study Protocol

#### Participant Selection and Data Collection

We identified patients with an order for DEX as receiving DEX in the ED by registry query, which was verified by review of the EHR. We excluded patients who did not actually receive DEX, as well as duplicate patients in the registry. All measurement and clinical data were gathered from the EHR using a standardized data collection form (created a priori), collated into an Excel 2016 (Microsoft Corporation, Redmond, WA, 2016) data management file, and exported to SPSS version 26, 2019 (IBM Corporation, Armonk, NY,) for management and data analysis. Prior to analysis, we checked the database for out-of-range and implausible values, and rechecked data as needed in the EHR to ensure accuracy. Baseline characteristics included the following: age; gender; race; body mass index; pre-existing comorbid conditions; disposition data; initial vital signs in the ED; and select laboratory values. Comorbid conditions were dementia, diabetes mellitus, cirrhosis, heart failure, end-stage renal disease, chronic obstructive pulmonary disease, immunosuppression, malignancy, alcohol abuse, and psychiatric illness (ie, schizophrenia, bipolar disorder, major depression, or generalized anxiety disorder). Laboratory values included lactate, creatinine, bilirubin, platelets, hemoglobin, and blood gases. The ED process of care variables included length of stay, vasopressor use, and need for mechanical ventilation.

We collected all DEX-related data in the ED including the following: indication for its use (per clinician documentation in the ED); time from ED arrival to order and time from order to drug administration; duration of use in the ED; dosing; and mental status (Richmond Agitation-Sedation Scale [RASS[ or Glasgow Coma Scale [GCS] at initiation. Additionally, we collected vital signs at initiation and their lowest values during drug infusion, and the number of patients in whom DEX was stopped in the ED, as well as co-administered analgesics and sedatives in the ED.

We collected details on adverse events and the treatment variables surrounding adverse events. The primary adverse events of interest included the incidence of hypotension and bradycardia. Similar to a prior large, randomized trial, hypotension was defined as a systolic blood pressure <80 millimeters mercury (mm Hg), a diastolic blood pressure <50 mm Hg, or > 30% decrease from baseline (systolic, diastolic, or mean arterial pressure). 3 Bradycardia was also defined based on prior trials, and included a heart rate < 40 beats per minute, < 60 beats per minute, or > 30% decrease from baseline.^3^,^4^ We also collected data regarding the need for vasoactive medications or fluid boluses after DEX initiation. If vasoactive medications or fluid boluses were given prior to DEX inititation, this was not counted as event secondary to DEX use. Finally, the cessation of DEX due to an adverse event was obtained from clinician documentation, and determined in the following manner: cessation due to hypotension and/or bradycardia, as defined in adverse events; or if cessation occurred due to inadvertent extubation.

An a priori subgroup of interest were the patients requiring mechanical ventilation in the ED.

#### Outcomes

We analyzed all included patients for characterization of DEX in the ED. The primary outcome of interest was the incidence of hypotension and bradycardia related to DEX use. Other clinical outcomes of interest included the incidence of acute brain dysfunction on ICU day 1 (delirium and coma), ventilator-, ICU- and hospital-free days, and hospital mortality. Coma was defined as having a RASS of −4 or −5 for every measurement while in the ICU. “Free” days account for both time (ie, duration of ventilation or lengths of stay) and mortality and are indexed to study day 28. In participants who survived 28 days, “-free” days are defined as 28 minus duration of ventilation (ventilator-free days) or length of stay (ICU- and hospital-free days). Participants who did not survive 28 days were assigned zero “-free” days.

#### Analysis

Patient characteristics are reported using descriptive statistics, including mean (standard deviation [SD]) and median (interquartile range [IQR]), and frequency distributions. We compared continuous variables using independent samples *t*-test or Mann-Whitney *U* test, whereas categorical variables were compared using chi-square test or Fisher’s exact test. We assessed the normality of the data by inspection of Q-Q plots and the Kolmogorov-Smirnov test.

For the purposes of this analysis, the primary outcome of adverse events was a composite outcome of hypotension or bradycardia. To examine for potential variables associated with adverse events we used a multivariable logistic regression model. In anticipation of a small number of events, we chose a parsimonious model and followed recommendations to select covariates a priori. 11 We therefore selected the following predictors for the model: 1) vasopressor infusion in the ED; 2) DEX duration in the ED; 3) heart rate at initiation of DEX; and 4) mechanical ventilation use in the ED. These variables were chosen for the following reasons: 1) Patients in shock may be more prone to experience hypotension related to DEX use; 2) a longer duration of use would allow greater time for adverse events to occur; 3) a lower baseline heart rate may lead to a higher incidence of bradycardia; and 4) mechanically ventilated patients are sicker and typically require more sedation than non-intubated patients, therefore predisposing them to a higher complication rate.

All tests were two-tailed with an alpha of 0.05 for statistical significance. As the study design is a retrospective cohort study over a fixed time frame, the sample size was limited to the number of patients receiving DEX during the course of routine care in the ED. Based on randomized trials examining DEX use in mechanically ventilated patients, we expected an adverse event rate ranging anywhere from 20–50%. 2–4 Assuming an estimated event (ie, composite adverse event) per covariable ratio of 10:1 necessary for multivariable logistic modeling, we assumed a sample size of 100 patients would be adequate to describe DEX use in the ED and explore factors associated with adverse events, in a hypothesis-generating multivariable model.^12^,^13^

## RESULTS

**Figure 1:**
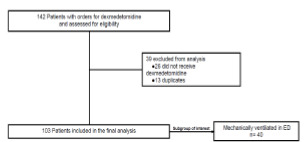
Flow diagram of included patients who had orders for dexmedetomidine. *ED*, emergency department.

A total of 103 patients were included in the study, and Figure 1 shows the study flow and final study population. Baseline characteristics are reported in Table 1. There was a statistical difference in peripheral oxygen saturation (mean [SD]) between patients experiencing an adverse event vs those who did not (93 [10] vs 96 [50], *P* = 0.018]. There were no other significant differences between patients experiencing an adverse event vs those who did not.

Dexmedetomidine-related variables are shown in Table 2. Acute respiratory failure, including mechanical ventilation (28.9%) and NIPPV (17.4%), was the most common indication for DEX, followed by control of agitation (14.9%) and anxiety (11.6%). The median starting dose in the ED was 0.4 mcg/kg/hour (0.2 – 0.4). However, variability in starting dose did exist, as 16 patients were started at a dose of 0.7 mcg/kg/hour or higher (3 patients ≥ 1.0 mcg/kg/hour). Median infusion rate remained at 0.4 mcg/kg/hour for the first four hours, and the highest median infusion rate was 0.7 (0.4 – 0.9), demonstrating that, overall, relatively low doses of DEX were used in the ED. Dexmedetomidine was stopped in the ED in 22 (18.2%) patients. Co-administered analgesics and sedatives included fentanyl (39.8%); ketamine (36.9%); midazolam (33%); lorazepam (31.1%); haloperidol (28.2%); and propofol (27.2%).

Adverse events and clinical outcomes are reported in Table 3. Fifty-four (52.4%) patients experienced the composite adverse event, with hypotension occurring in 41 patients (39.8%) and bradycardia occurring in 18 patients (17.5%). Patients experiencing an adverse event were given a fluid bolus (20.4% vs 3.7%, *P* <0.01) and vasoactive medications (12.2% vs 3.7%, *P* = 0.11) more frequently when compared to patients without an adverse event. Dexmedetomidine was stopped secondary to an adverse event in eight patients (7.8%). Clinical outcomes for patients experiencing an adverse event vs those in patients with no adverse event (mean [SD]), were as follows: ventilator-free days, (20.4 [10.5] vs 22.6 [8.7], *P* = 0.44); ICU-free days, (21.7 [8.1] vs 21.3 [8.4], *P* = 0.83),; and hospital-free days (18.5 [8.1] vs 17.5 [8.7], *P* = 0.53). Mortality among patients experiencing an adverse event when compared to those with no adverse event was 10.2% vs 9.3%, *P* = 0.87.

Table 4 shows the multivariable logistic regression analysis for predictors of the composite primary outcome. Duration of DEX use in the ED was associated with an increased risk for hypotension or bradycardia (adjusted odds ratio [aOR], 1.004; 95% CI, 1.001, 1.008), while vasopressor infusion in the ED was associated with a decrease risk (aOR, 0.21; 95% CI, 0.05, 0.82).

Details regarding the mechanically ventilated subgroup are provided in supplemental tables S2–4. Overall, the dosing characteristics and adverse events experienced by mechanically ventilated patients were similar to the entire cohort.

## DISCUSSION

As sedation and pain control are cornerstone therapies provided in the ED, and with the increase in use of DEX, information regarding its use in the ED is critical before quality improvement or future research can occur. The current study provides some new information regarding DEX use in the ED and builds on prior work by examining this agent in the ED domain.

With respect to our first objective, DEX is used for diverse indications in the ED, and most commonly for patients with respiratory failure. This is congruent with prior work and facilitated by DEX’s analgesic and sedative properties, without suppression of respiratory drive. The co-administration of other sedatives and analgesics was common, and could be driven by the known limitations of DEX, such as slower onset of action. There was a delay in administration of DEX (156 minutes) and relatively static dosing in the ED. This is likely driven by the lack of DEX in the ED (ie, ordered from pharmacy), as well as the institutional protocol, which called for no titration (in non-intubated patients) or physician-ordered titration (in mechanically ventilated patients). Going forward, areas for potential improvement could be as follows: 1) earlier identification of patients who may benefit from DEX, given the 2.5 hours of elapsed time from patient arrival to order; and 2) titrated dosing if DEX is tolerated, yet sedation goals have not been achieved.

Our most important finding relates to the adverse events experienced by ED patients given DEX. Prior work in difficult-to-sedate patients (n = 13) stated that DEX “is not safe in the ED setting.”^14^ Our results would suggest otherwise, and demonstrate that an ED-based DEX protocol can be effectively implemented. While adverse events were relatively common, the event rate for DEX use is congruent with that experienced in large randomized trials. 2–4 Also, when placed in the context of the reported incidence of hypotension with midazolam (11.6% to 55.7%) and propofol (13.4% to 52.4%) described in the literature, our results further suggest that DEX compares favorably in the ED setting.^3^,^15^,^16^ Furthermore, in only eight patients (7.8%) did physicians stop DEX due to an adverse event, suggesting that while hypotension and bradycardia were relatively common, these events were clinically well tolerated as judged by the treating team. Patients experiencing adverse events did require more intensive therapy in the ED, as demonstrated by the administration of more fluid boluses and vasoactive medications.

There was no statistical difference in patient-centered clinical outcomes between patients experiencing an adverse event when compared to those who did not. However, we urge caution in interpreting these clinical outcome data, given the small sample size. Contrary to our rationale for including vasopressors in the multivariable model, vasopressor infusion in the ED was associated with a lower chance for adverse events. It is possible that vasopressor titration reduced the risk of reported hypotension. While our study lacks granular detail on pressor requirements during DEX infusion, this finding is congruent with prior work showing that DEX is well tolerated in patients with shock. 17 A potentially important finding is the fact that duration of DEX exposure in the ED was associated with adverse events. While we lack specific detail on the exact timing of events, these data suggest the need for ongoing diligent monitoring for safety while DEX is being used in the ED.

Finally, in our subgroup of mechanically ventilated patients, the dosing of DEX and adverse events were comparable to non-intubated patients. While no definitive conclusions can be drawn from this small sample size, our findings suggest that DEX use in the ED could be a viable option going forward.

## LIMITATIONS

This study has several limitations. This was a retrospective, single-center study that carries with it all of the limitations of that design, including limits with respect to generalizing these results to other centers, especially those where DEX use is infrequent in both the ED and ICU. While to our knowledge this is the largest ED-based DEX study to date, the small sample size limits any conclusions that can be drawn from these data. We further emphasize that point with respect to the subanalysis with an even smaller sample size and commensurate power limitations secondary to that. Due to an overall lack of sedation depth documentation, we cannot comment on the efficacy of DEX use in the ED. Future studies will need to assess for sedation depth, pain control, and anxiolysis in a much more granular fashion.

We defined our adverse events based on prior work from randomized trials on DEX in mechanically ventilated patients. While our adverse event rate was congruent with prior work, had our definition differed, the incidence of hypotension and bradycardia experienced in the ED could be lower than our current definition. This is especially important when considering that only eight patients had their DEX infusion stopped because of an adverse event. We also do not have details on why DEX was stopped outside of adverse events. It is possible that DEX was stopped because of inefficacy, or improving clinical trajectory. Due to the study design, it is impossible to ascribe causation for the adverse events, as multiple agents were used in addition to DEX, and we can only describe associations. Finally, due to the overall low event rate and small sample size, the results of our multivariable model should be considered exploratory and hypothesis-generating at this point.

## CONCLUSION

Dexmedetomidine is most commonly administered in the ED for patients with acute respiratory failure (ie, those requiring mechanical ventilation or NIPPV). While adverse events are relatively common, they are of questionable clinical significance. Our results suggest that dexmedetomidine can be incorporated effectively into clinical care in the ED and be a viable option for analgesia, anxiolysis, and sedation in ED patients, similar to its role in the ICU.

## Supplementary Information









## Figures and Tables

**Table 1:** Characteristics of included study participants.

Baseline characteristics	All subjects (n = 103)	No adverse event (n = 54)	Adverse event (n = 49)	*P*
Age (years)	54 (37–65)	55 (42–65)	54 (35–65)	0.692
Female, n (%)	39 (32.2)	23 (42.6)	16 (32.7)	0.299
BMI	27.0 (22.4–35.0)	29.1 (23.8–35.0)	25.1 (21.1–35.8)	0.248
Race, n (%)				
Black	52 (43.0)	27 (50.0)	25 (51.0)	0.918
White	51 (42.1)	27 (50.0)	24 (49.0)	
Comorbidities, n (%)				
Dementia	3 (2.5)	1 (1.9)	2 (4.1)	0.502
Diabetes mellitus	31 (25.6)	17 (31.5)	14 (28.6)	0.748
Cirrhosis	7 (5.8)	4 (7.4)	3 (6.1)	0.796
Heart failure	16 (13.2)	10 (18.5)	6 (12.2)	0.380
ESRD	5 (4.9)	4 (7.4)	1 (2.0)	0.206
COPD	22 (18.2)	14 (25.9)	8 (16.3)	0.235
Alcohol abuse	27 (22.3)	16 (29.6)	11 (22.4)	0.408
Illicit drug abuse	29 (24.0)	17 (31.5)	12 (24.5)	0.431
Psychiatric^a^	16 (13.2)	6 (11.1)	10 (20.4)	0.193
Disposition Data, n (%)				
Admit Location				0.472
ICU	97 (80.2)	50 (92.6)	47 (95.9)	
Floor	6 (5.0)	4 (7.4)	2 (4.1)	
Temperature (^o^C)	36.7 (36.4–37.1)	36.6 (36.3–37.0)	36.7 (36.5–37.2)	0.164
Heart rate (bpm)	107 (23)	104 (23)	109 (22)	0.249
Respiratory Rate (bpm)	23 (7)	23 (7)	23 (7)	0.684
Systolic pressure (mm Hg)	145 (30)	143 (26)	146 (33)	0.646
Diastolic pressure (mm Hg)	89 (22)	89 (21)	88 (23)	0.848
Peripheral oxygen saturation (%)	94 (8)	96 (50)	93 (10)	0.018
Lactate (mmol/L)	2.3 (1.4–3.6)	2.3 (1.4–3.5)	2.2 (1.3–4.7)	0.900
Creatinine (mg/dL)	1.0 (0.8–1.3)	1.1 (0.8–1.3)	1.0 (0.7–1.2)	0.289
Bilirubin (mg/dL)	0.4 (0.3–0.6)	0.4 (0.3–0.6)	0.4 (0.3–0.8)	0.616
pH (n = 78)	7.31 (0.13)	7.31 (0.11)	7.30 (0.14)	0.560
Partial pressure arterial oxygen (n=34)	150 (76)	144 (61)	157 (93)	0.628
Partial pressure arterial or venous carbon dioxide (n = 78)	48 (17)	45 (11)	52 (21)	0.086
SOFA score	1.0 (0–4.0)	1.0 (0–3.0)	1.0 (1.0–4.0)	0.697
ED process of care variables				
Length of stay (hours)	7.1 (4.7–9.6)	6.7 (4.5–8.7)	7.9 (5.2–10.3)	0.101
Vasopressor infusion, n (%)	14 (11.6)	4 (7.4)	10 (20.4)	0.055
Mechanically ventilated, n (%)	40 (33.1)	24 (44.4)	16 (32.7)	0.220

a Psychiatric if diagnosed with schizophrenia, bipolar, major depression, or generalized anxiety disorder

Continuous variables are reported as mean (standard deviation) and median (interquartile range).

*BMI*, body mass index; *ESRD*, end-stage renal disease; *COPD*, chronic obstructive pulmonary disease; *ICU*, intensive care unit; *C*, Centigrade; *bpm*, beats per minute; *bpm*, breaths per minute; *mm Hg*, millimeters mercury; *mmol/L*, millimoles per liter; *mg/dL*, milligrams per deciliter; *SOFA*, sequential organ failure assessment; *ED*, emergency department.

**Table 2:** Dexmedetomidine dosing and sedation characteristics.

Variable	All subjects (n = 103)		No adverse event (n = 54)		Adverse event (n = 49)		*P*	
Indication for dexmedetomidine, n (%)^*^							0.847	
Procedural sedation	4 (3.3)		2 (3.7)		2 (4.1)			
Alcohol withdrawal	9 (7.4)		5 (9.3)		4 (8.2)			
Anxiolysis	14 (11.6)		5 (9.3)		9 (18.4)			
Psychosis/agitation	18 (14.9)		10 (18.5)		8 (16.3)			
Facilitation of NIPPV	21 (17.4)		10 (18.5)		11 (2.4)			
Sedation for mechanical ventilation	35 (28.9)		21 (38.9)		14 (28.6)			
Other	2 (1.7)		1 (1.9)		1 (2.0)			
Time from ED arrival to order (minutes)	156 (64 – 317)		170 (73 – 317)		136 (42 – 333)		0.722	
Time from order to administration (minutes)	26 (11 – 55)		42 (16 – 60)		21 (9 – 32)		0.021	
Duration of dexmedetomidine in ED (minutes)	139 (74 – 211)		122 (69 – 207)		164 (96 – 240)		0.041	
Starting dose in ED (mcg/kg/hour)	0.4 (0.2 – 0.4)		0.4 (0.2 – 0.5)		0.4 (0.2 – 0.4)		0.267	
RASS at initiation of dexmedetomidine (n= 29)	1 (0 – 3)		1 (−1 to 3)		1 (0 – 2)		0.811	
GCS at initiation of dexmedetomidine (n= 40)	13 (10 – 15)		13 (11 – 15)		13 (9 – 14)		0.366	
Co-administered analgesics and sedatives, n (%)							0.248	
Fentanyl	41 (39.8)		23 (42.6)		18 (36.7)			
Propofol	28 (27.2)		16 (29.6)		12 (24.5)			
Midazolam	34 (33.0)		17 (31.5)		17 (34.7)			
Ketamine	38 (36.9)		18 (33.3)		20 (40.8)			
Lorazepam	32 (31.1)		15 (27.8)		17 (34.7)			
Haloperidol	25 (24.3)		14 (25.9)		11 (22.4)			
Vital signs	At initiation	Lowest during infusion	At Initiation	Lowest during infusion	At Initiation	Lowest during infusion	At initiation	Lowest during infusion
Heart rate (bpm)	105 (23)	86 (21)	102 (21)	91 (22)	108 (25)	81 (19)	0.163	0.010
Respiratory rate (bpm)	23 (7)	20 (18)	24 (7)	20 (6)	23 (7)	18 (5)	0.697	0.051
Systolic blood pressure (mm Hg)	140 (29)	112 (25)	141 (27)	124 (21)	138 (32)	99 (22)	0.606	<0.001
Diastolic blood pressure (mm Hg)	85 (24)	68 (18)	86 (20)	77 (16)	84 (27)	58 (16)	0.740	<0.001
Mean arterial pressure (mm Hg)	101 (24)	82 (19)	102 (21)	92 (16)	100 (27)	71 (16)	0.796	<0.001
Dexmedetomidine infusion stopped in ED, n (%)^a^	22 (18.2)		11 (20.4)		11 (22.4)		0.797	

a Eighteen patients were documented as having an additional secondary indication for dexmedetomidine use.

*NIPPV*, non-invasive positive pressure ventilation; *ED*, emergency department; *mcg/kg/hour*, micrograms/kilogram/hour; *RASS*, Richmond Agitation-Sedation Scale; *GCS*, Glasgow Coma Scale; *bpm*, beats per minute; bpm, breaths per minute; *mm Hg*, millimeters mercury.

**Table 3:** Adverse events and clinical outcomes.

Variable	All subjects (n = 103)
Hypotension, n (%)	41 (39.8)
SBP <80 mm Hg	8 (7.8)
DBP <50 mm Hg	14 (13.6)
>30% decrease from baseline*	19 (18.4)
Bradycardia, n (%)*	
<60 bpm	18 (17.5)
<40 bpm	0 (0.0)
Vasoactive medication given after dexmedetomidine initiated, n (%)	8 (7.8)
Fluid bolus given after dexmedetomidine initiation, n (%)	12 (11.7)
Cessation of dexmedetomidine due to adverse event, n (%)	8 (7.8)
Starting dose in ED (mcg/kg/hour)	0.4 (0.2 – 0.4)
Acute brain dysfunction on day 1 ICU, n (%)	
Delirium	63 (61.2)
Coma	0
ICU-free days**	21.5 (8.2)
Hospital-free days	18.0 (8.4)
Hospital mortality, n (%)	10 (9.7)

* Refers to a decrease in systolic, diastolic, or mean arterial pressure.

** Refers to the 97 patients admitted to the intensive care unit from the emergency department.

Continuous variables are reported as mean (standard deviation) and median (interquartile range).

*SBP*, systolic blood pressure; *DBP*, diastolic blood pressure; *bpm*, beats per minute; *ED*, emergency department; *mcg/kg/hour*, micrograms/kilogram/hour; *ICU*, intensive care unit.

**Table 4:** Multivariable logistic regression analysis with a composite of hypotension and bradycardia as the dependent variable.

Variables	aOR	95% CI	Standard error	P
Vasopressor infusion in the ED	0.21	0.05 – 0.82	0.70	0.025
Dexmedetomidine duration in the ED	1.004	1.001 – 1.008	0.01	0.022
Heart rate at initiation of dexmedetomidine	1.01	0.99 – 1.03	0.01	0.238
Mechanical ventilation in the ED	1.63	0.60 – 4.40	0.51	0.341

*ED*, emergency department; *aOR*, adjusted odds ratio; *CI*, confidence interval.
