# Supplementary material for: The Use of Dexmedetomidine in the Emergency Department: A Cohort Study
Source: West J Emerg Med. 2021 Aug 22;22(5):1202–9. doi: 10.5811/westjem.2021.4.50917 (PMC8463063; doi:10.5811/westjem.2021.4.50917)
Supplement: Supplementary file 2 [file wjem-22-1202-s002.docx]

**Supplemental Table 2**. Characteristics of mechanically ventilated patients.

| **Baseline Characteristics** | **All Subjects**  **(n = 40)** |
| --- | --- |
| Age (years) | 53 (33-63) |
| Female, n (%) | 16 (40.0) |
| BMI | 29.6 (23.5-36.6) |
| Race, n (%) |  |
| Black | 16 (40.0) |
| White | 24 (60.0) |
| Comorbidities, n (%) |  |
| Dementia | 0 (0) |
| Diabetes Mellitus | 10 (25.0) |
| Cirrhosis | 1 (2.5) |
| Heart Failure | 16 (13.2) |
| ESRD | 1 (2.5) |
| COPD | 8 (20.0) |
| Immunosuppression | 14 (4) |
| Malignancy | 41 (11) |
| Alcohol Abuse  Illicit drug abuse | 11 (27.5)  16 (40.0) |
| Psychiatric^a^ | 6 (15.0) |
| Temperature (^o^C) | 36.5 (35.8-37.0) |
| Heart Rate (bpm) | 105 (25) |
| Respiratory Rate (bpm) | 22 (7) |
| Systolic Pressure (mmHg) | 139 (30) |
| Diastolic Pressure (mmHg) | 85 (20) |
| Peripheral Oxygen Saturation (%) | 94 (9) |
| Lactate (mmol/L) | 2.4 (1.5-3.5) |
| Creatinine (mg/dL) | 1.1 (0.8-1.3) |
| Bilirubin (mg/dL) | 0.3 (0.2-0.6) |
| pH (n=34) | 7.30 (0.12) |
| Partial pressure arterial oxygen (n= 21) | 162 (79) |
| Partial pressure arterial carbon dioxide (n= 34) | 47 (14) |
| SOFA | 2.5 (1.0-4.0) |
| ED Process of Care Variables |  |
| Length of Stay (hours)  Duration of ED mechanical ventilation (hours) | 5.8 (3.9-5.4)  4.0 (2.2-5.4) |
| Vasopressor Infusion, n (%) | 11 (27.5) |
| Indication for mechanical ventilation, n (%)  Trauma  Altered mental status  Sepsis  Sudden cardiac arrest  Overdose  COPD  CHF  Other | 12 (30.0)  10 (25.0)  4 (10.0)  4 (10.0)  3 (7.5)  2 (5.0)  1 (2.5)  4 (10.0) |

BMI = body mass index, ESRD = end-stage renal disease, COPD = chronic obstructive pulmonary disease, ICU= Intensive Care Unit, SOFA = Sequential Organ Failure Assessment

^a^Psychiatric if diagnosed with schizophrenia, bipolar, major depression, or generalized anxiety disorder

Continuous variables are reported as mean (standard deviation) and median (interquartile range).
