# Supplementary material for: The Use of Dexmedetomidine in the Emergency Department: A Cohort Study
Source: West J Emerg Med. 2021 Aug 22;22(5):1202–9. doi: 10.5811/westjem.2021.4.50917 (PMC8463063; doi:10.5811/westjem.2021.4.50917)
Supplement: Supplementary file 3 [file wjem-22-1202-s003.docx]

**Supplemental Table 3**. Dexmedetomidine sedation characteristics for mechanically ventilated patients

| **Variable** | **All Subjects**  **(n = 40)** | |
| --- | --- | --- |
| Time from ED arrival to order (minutes) | 115 (68 – 309) | |
| Time from order to administration (minutes) | 26 (10 – 54) | |
| Duration of dexmedetomidine in ED (minutes) | 95 (45 – 167) | |
| Starting dose in ED (mcg/kg/hour) | 0.4 (0.2 – 0.7) | |
| RASS at initiation of dexmedetomidine (n= 12) | 1 (-3 to 2) | |
| GCS at initiation of dexmedetomidine (n= 13) | 9 (3 – 15) | |
| Vital signs  Heart rate (bpm)  Respiratory rate (bpm)  Systolic blood pressure (mmHg)  Diastolic blood pressure (mmHg)  Mean arterial pressure (mmHg) | At Initiation  98 (23)  22 (5)  130 (30)  78 (22)  94 (22) | Lowest during infusion  83 (21)  19 (5)  108 (30)  65 (20)  78 (21) |
| Dexmedetomidine infusion stopped in ED, n (%) | 10 (25.0) | |
| Co-administered analgesics and sedatives, n (%)  Fentanyl  Propofol  Midazolam  Ketamine  Lorazepam  Haloperidol | 33 (82.5)  24 (60.0)  18 (45.0)  15 (37.5)  3 (7.5)  3 (7.5) | |

EM= emergency medicine, CCM= critical care medicine, ED = emergency department, RASS = Richmond Agitation-Sedation Scale, GCS= Glasgow Coma Scale

Continuous variables are reported as mean (standard deviation) and median (interquartile range).
