# Supplementary material for: The Use of Dexmedetomidine in the Emergency Department: A Cohort Study
Source: West J Emerg Med. 2021 Aug 22;22(5):1202–9. doi: 10.5811/westjem.2021.4.50917 (PMC8463063; doi:10.5811/westjem.2021.4.50917)
Supplement: Supplementary file 4 [file wjem-22-1202-s004.docx]

**Supplemental Table 4**. Adverse events and clinical outcomes among mechanically ventilated patients

| **Variable** | **All Subjects**  **(n = 40)** |
| --- | --- |
| Hypotension, n (%)  SBP Complication  DBP Complication  >30% Complication | 14 (35.0)  8 (20.0)  4 (10.0)  2 (5.0) |
| Bradycardia, n (%)* | 3 (7.5) |
| Heart rate < 40 bpm, n (%) | 0 (0.0) |
| Inadvertent extubation, n (%) | 1 (2.5) |
| Vasoactive medication given after dexmedetomidine initiated, n (%) | 6 (15.0) |
| Fluid bolus given after dexmedetomidine initiation, n (%) | 6 (15.0) |
| Cessation of dexmedetomidine due to adverse event, n (%) | 5 (12.5) |
| Starting dose in ED (mcg/kg/hour) | 0.4 (0.2 – 0.7) |
| Dexmedetomidine continued in the ICU, n (%) | 23 (57.5) |
| Acute brain dysfunction on day 1 ICU, n (%)  Delirium  Coma | 25 (62.5)  0 |
| Ventilator-free days | 21.4 (10.2) |
| ICU-free days | 19.8 (8.9) |
| Hospital-free days | 16.5 (9.3) |
| Hospital mortality, n (%) | 4 (10.0) |

SBP= systolic blood pressure, DBP= diastolic blood pressure, ICU= intensive care unit

^*^Defined as heart rate < 60 or more than a 30% decrease from baseline.

Continuous variables are reported as mean (standard deviation) and median (interquartile range).
